# Pathogenesis of Shoulder Calcific Tendinopathy

**DOI:** 10.3390/ijms27052178

**Published:** 2026-02-26

**Authors:** Rami Kaplan, Micaela Berni, Laura Caliogna, Greta Dei Rossi, Camilla Torriani, Eugenio Jannelli, Mario Mosconi, Federico Alberto Grassi, Gianluigi Pasta

**Affiliations:** 1 Department of Orthopedics, University of Cattolica Del Sacro Cuore, 00168 Rome, Italy; rami.kaplan01@icatt.it; 2 Orthopedics and Traumatology Clinic, IRCCS Policlinico San Matteo Foundation, 27100 Pavia, Italy; m.berni@smatteo.pv.it (M.B.); l.caliogna@smatteo.pv.it (L.C.); e.jannelli@smatteo.pv.it (E.J.); m.mosconi@smatteo.pv.it (M.M.); f.grassi@smatteo.pv.it (F.A.G.); 3 Department of Mechanical Engineering (DMEC), Politecnico di Milano, 20156 Milano, Italy; greta.deirossi@polimi.it; 4 Department of Public Health, Experimental and Forensic Medicine, University of Pavia, 27100 Pavia, Italy; camilla.torriani01@universitadipavia.it; 5 Department of Clinical, Surgical, Diagnostic and Pediatric Sciences, University of Pavia, 27100 Pavia, Italy

**Keywords:** calcific tendinopathy, shoulder, pathogenesis

## Abstract

Shoulder calcific tendinopathy is a common condition affecting adults and is has a higher incidence in women. This condition is due to a multifactorial process and is characterized by the deposition of hydroxyapatite crystals in the rotator cuff tendons. The disease shows a phenotypic transformation of tenocytes into chondrocyte-like cells, likely caused by metabolic and inflammatory changes and mechanical stress. Risk factors promoting this pathology include hyperlipidemia, advanced age, diabetes, female gender, and thyroid dysfunction. Recent studies highlight that metalloproteinases, oxidative stress, inflammatory mediators, bone morphogenetic proteins (BMPs), genetic and post-transcriptional alterations play a significant role in the pathogenesis of the disease. New therapeutic strategies are currently available that aim to modulate inflammation, osteogenic differentiation, and calcium homeostasis, showing promising results, especially in preclinical models. The aim of this review is to explore the different pathogenetic mechanisms and highlight future therapeutic developments for the treatment of shoulder calcification.

## 1. Introduction

Shoulder tendon calcification is a prevalent condition that is routinely encountered in clinical practice, primarily affecting people between the ages of 30 and 60 [1]. Studies report that it can be present in anywhere from 6.8 to 40% of patients experiencing shoulder pain, and from 2.7 to 20% of asymptomatic individuals [1,2,3]. Women are more often affected than men, and the reason for this is still unknown [1]. However, this could be due to a combination of hormonal differences and a higher prevalence of endocrine disorders in women [3]. Due to its prevalence, these pathology and etiology are still much studied.

The first study dates back to 1931 when Codman and Akerson discover that rotator cuff tears are associated with progressive degenerative changes, and observe tendon fraying, thinning, and granulation, suggesting that the pathology developed over time due to chronic mechanical stress and poor vascular supply [4]. Subsequently, Sandström publishes the first comprehensive clinical and radiographic description of calcific tendinopathy and coins the term “peritendinitis calcarea”. Moreover, he hypothesizes that localized ischemia in poorly vascularized tendon regions leads to tissue necrosis and subsequent calcium deposition [5]. Following Sandström’s theory, Mohr and Bilger develop a model in which the shoulder calcification begins with the necrosis of tenocytes, accompanied by intracellular accumulation of calcium, typically forming small, round concretions known as microspheroliths or psammomas [6]. Finally, bringing together all these hypotheses the most comprehensive model of calcific tendinopathy is Uhthoff’s theory [7]. The theory describes shoulder calcification as a three-stage process: pre-calcific stage, calcific stage, and post-calcific stage [7].

The pre-calcific stage is defined by fibrocartilaginous metaplasia of tenocytes, when normal tendon cells are transformed into chondrocytes. This adverse differentiation of tendon cells may be due to an overexpression of ProstaGlandin E2 (PGE2), mainly released by tenocytes and resident tendon fibroblasts in response to increased mechanical load [8]. Although it appears asymptomatic, this stage creates the necessary conditions for mineralization [9].

The calcific stage, instead, is subdivided into three distinct phases: the formative phase, the resting phase and the resorptive phase. During the formative phase, calcium hydroxyapatite crystals accumulate via cell-mediated processes, forming deposits. Following, during the resting phase, the calcifications become stable and encapsulated, typically causing little to no symptoms. Finally, the resorptive phase is characterized by the infiltration of macrophages and giant cells, breaking down the deposits into a soft, toothpaste-like material that may leak into surrounding tissues, thereby severe pain and acute symptoms [10].

Lastly, the post-calcific stage is about the tendon-healing phase that occurs after calcium resorption: the fibroblasts infiltrate the area and produce type III collagen, which is gradually replaced by type I collagen to restore normal tendon structure. While symptoms often resolve, residual fibrovascular or scar tissue may persist, potentially impairing tendon function [11]. Regulatory T cells (Tregs) are known to support tendon repair by suppressing inflammation and promoting the transition of macrophages from pro-inflammatory M1 phenotype to a reparative M2 phenotype, thereby enhancing fibroblast activity and collagen remodeling [12].

Moreover, several studies have reported alterations in various molecular and genetic factors associated with shoulder calcification [13,14,15]. For example, some metalloproteinases (MMP-9 and MMP-13) are involved in extracellular matrix (ECM) breakdown, while Fatty Acid Binding Protein (FABP4) is associated with the increased expression of inflammatory cytokines in tendinopathy.

The aim of this review is to explore the different mechanisms of pathogenesis, including cellular, molecular and genetic factors, to summarize the current studies behind this pathological condition and to suggest future therapies development for shoulder calcification treatments.

## 2. Results

Calcific tendinopathy of the shoulder appears to be multifactorial; however, a central pathological event appears to be the phenotypic transformation of tenocytes. The causes of this transformation remain unclear [16]. To better understand them we conducted literature search using PubMed, Medline, Scopus and Web of Science database.

One hypothesis, proposed by Uhthoff et al. [7,17], is that tenocytes undergo metaplasia into chondrocyte-like cells, potentially driven by mechanical compression, which induces fibrocartilaginous changes in the tendon, especially in the overused supraspinatus tendon. This is supported by the upregulation of cartilage-related genes and the presence of chondroid cells in degenerative lesions, although overuse alone cannot explain all cases [7,17]. Another theory suggests that these chondrocyte-like cells may originate from tendon-resident stem cells, which have the capacity to differentiate into cartilage [18]. Additional debate persists on whether calcific tendinopathy represents a degenerative phenomenon leading to dystrophic calcification or, as Uhthoff proposed, a reparative process occurring in viable, well-vascularized tissue.

### 2.1. Risk Factors

Several systemic, metabolic, and demographic factors are implied in the pathogenesis of shoulder calcific tendinopathy.

Diabetes mellitus is a well-recognized risk factor for shoulder calcific tendinopathy. Hyperglycemia-associated alterations, including increased free radical production, oxidative stress, accumulation of advanced Glycation End Products (AGEs), dysregulated expression of inflammatory factors, and metabolic changes in the local microenvironment, can modify tendon structure and affect vascular supply, thereby promoting fibrocartilaginous transformation and subsequent calcification [19]. Large cohort studies show that individuals with diabetes exhibit a 27% higher risk of developing shoulder calcific tendinopathy over eight years, highlighting diabetes as a major predisposing factor. These studies identify older age and female sex as additional risk factors [20].

Immediately following diabetes, endocrine disorders, particularly thyroid dysfunction, represent another important risk factor. Hypothyroidism is frequently observed among patients with calcific tendinitis and may contribute to disease development, as thyroxine plays a crucial role in collagen synthesis and extracellular matrix metabolism. Consequently, hypothyroidism may lead to altered tendon composition or impaired remodeling, increasing the risk of calcification in the shoulder region [21].

Age-related differences further emerge, with older individuals displaying an increased susceptibility to shoulder calcific tendinopathy. Sex-specific differences are also observed, with women showing an increased susceptibility to shoulder calcific tendinitis, particularly in the presence of hyperlipidemia, which markedly double the risk compared with women without lipid disorders. In contrast, no significant association is observed in men with hyperlipidemia, suggesting a possible sex-dependent interaction in disease susceptibility [22].

Although occupational exposure and repetitive movements are often considered potential risk factors, epidemiological evidence remains inconsistent. A population-based study by Sansone et al. conducted on female cashiers did not identify a significant increase in risk, suggesting that calcification may arise predominantly from factors other than repetitive occupational activity [23].

An overview of the main predisposing conditions and associated epidemiological evidence is provided in Table 1.

### 2.2. Genetic Factors

Beyond common metabolic and demographic risk factors, a subset of hereditary and molecular disorders affecting mineral metabolism, extracellular pyrophosphate homeostasis, and post-transcriptional regulation is implicated in the development of shoulder calcific tendinopathy.

Calcium Pyrophosphate Dihydrate Deposition (CPPD) may contribute to, or mimic, calcific tendinopathy and is tightly regulated by extracellular inorganic pyrophosphate (PPi) homeostasis. The progressive ankilosis homolog (ANKH) gene encodes a transmembrane protein involved in the transport of PPi from the intracellular (plasma) to the extracellular compartment. Gain-of-function mutations in ANKH are associated with both familial and sporadic forms of CPPD. Familial CPPD includes conditions such as Chondrocalcinosis 2 (CCAL2), an autosomal dominant disorder linked to chromosome 5p. Certain mutations, including Proline-to-Leucine substitution at position 5 (P5L), are shown to increase type X collagen expression, suggesting a role in chondrocyte maturation and cartilage-like differentiation [15].

Hypophosphatasia is also proposed as a potential cause of shoulder calcification. This hereditary disorder results from reduced serum alkaline phosphatase activity (ALP) due to mutations in the gene encoding the tissue-nonspecific isoenzyme of alkaline phosphatase (TNSALP), located on chromosome 1. Both autosomal dominant and recessive forms are described, with more than 260 pathogenic TNSALP mutations identified to date. The condition is characterized by hypercalcemia and skeletal abnormalities and may present with calcification around joints and ligaments [24].

Alkaptonuria is a rare autosomal recessive disorder caused by mutations in the homogentisate 1,2-dioxygenase (HGD) gene, leading to the accumulation of homogentisic acid in collagen-rich tissues. This results in ochronosis, characterized by pigment deposition, degeneration of connective tissue, and disruption of collagen cross-linking. These pathological changes may manifest as calcification in tendons and ligaments, making alkaptonuria a potential contributor to shoulder calcification [24].

Pseudoxanthoma elasticum (PXE) is another rare autosomal recessive disorder caused by mutations in the ATP-binding cassette subfamily C member 6 (ABCC6) gene. These mutations lead to reduced extracellular PPi levels and increased tissue-nonspecific ALP activity, ultimately promoting ectopic soft tissue mineralization. Clinical studies report rotator cuff calcifications and enthesophytes in up to 78% of PXE patients, supporting its role as a risk factor for shoulder tendon calcification [25].

In addition to hereditary conditions, acquired post-transcriptional alterations are also implicated in calcific tendinopathy. An analysis of rotator cuff tendon samples from patients with shoulder calcification reveals abnormal localization of the RNA-binding protein RNA binding fox-1 homolog 2 (RBFOX2). While RBFOX2 is normally localized in the nucleus, where it regulates alternative splicing, it is predominantly detected in the cytoplasm of diseased tendons. This mislocalization is attributed to an alternative splicing event resulting in exon 10 skipping, which removes the nuclear localization signal. Consequently, RBFOX2 loses its ability to regulate the pre-mRNA splicing of target genes, such as chromodomain helicase DNA binding protein 2 (CHD2) and muscleblind-like splicing regulator 1 (MBNL1), both of which exhibit altered exon usage in calcified tendons [26].

The main genetic, hereditary, and molecular conditions associated with shoulder tendon calcification, along with their underlying mechanisms, are summarized in Table 2.

### 2.3. Cellular and Molecular Mechanisms

Multiple cellular and molecular mechanisms contribute to the pathological progression of calcific tendinopathy, with inflammation-driven dysregulation of tendon stem cell fate playing a central role. PGE2 is a key inflammatory mediator in tendon tissue and is shown to inhibit tendon stem cell proliferation while diverting their differentiation away from a tenogenic lineage toward a chondro-osteogenic phenotype. This shift is primarily mediated through the induction of bone morphogenetic protein-2 (BMP-2), which promotes ectopic calcification. Experimental evidence from *in vitro* studies using human tendon stem cell cultures demonstrates that, in the absence of BMP-2, the pathological effects of PGE2 are largely abolished, confirming BMP-2 as a critical downstream effector of PGE2 signaling [27].

**Figure 1:**
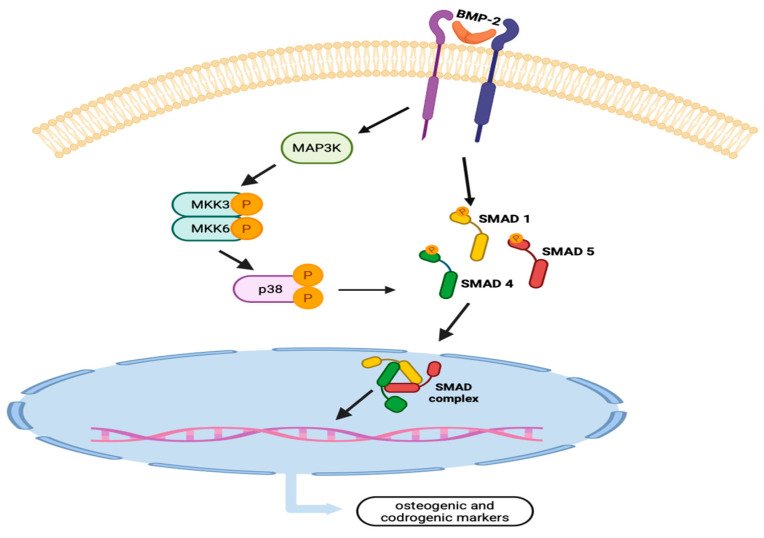
*BMP-2 pathway and its mechanism to activate osteogenic marker.* Abbreviations: BMP-2 (bone morphogenetic protein-2) MAP3K (Mitogen Activated Protein (MAP) kinase kinase kinase), MKK3 (Mitogen-activated protein kinase kinase 3), MKK6 (MAP kinase kinase 6), SMAD1 (mother against decapentaplegic homologs proteins 1), SMAD 5 (mother against decapentaplegic homologs proteins 5), SMAD 4 (mother against decapentaplegic homologs proteins 4).

BMP-2 exerts its osteogenic effects by binding to type I and type II bone morphogenetic protein receptors (BMPR-I and BMPR-II), which are serine/threonine kinase receptors. Receptor activation leads to phosphorylation of mother against decapentaplegic homologs proteins (Smads), intracellular signaling mediators, specifically Smad1 and Smad5, which subsequently associate with Smad4 and translocate into the nucleus to regulate the expression of osteogenic genes. In addition to the canonical Smad pathway, BMP-2 also activates the p38 mitogen-activated protein kinase (MAPK) pathway, which is essential for proper Smad1 phosphorylation and nuclear translocation; in its absence, osteogenic markers, such as osteocalcin (OCN), are not expressed, resulting in impaired matrix mineralization [28] (Figure 1).

Beyond inflammatory signaling, tendon homeostasis is also regulated by intrinsic circadian mechanisms. Tendon tissue exhibits an autonomous circadian rhythm that controls the expression of Gremlin-2, a BMP antagonist encoded by the Gremlin-2 gene (Grem2). Gremlin-2 expression peaks during the daytime, inhibiting BMP-induced Smad1/5 phosphorylation and osteogenic differentiation, whereas Smad1/5 activation is enhanced at night when Gremlin-2 levels decline. Disruption of this circadian regulation, due to aging or genetic alterations, leads to sustained BMP signaling and increased tendon calcification. Although these findings are derived from Achilles and tail tendons, they may also be relevant to shoulder tendons and could partly explain the higher prevalence of calcific tendinopathy in older individuals [28].

**Figure 2:**
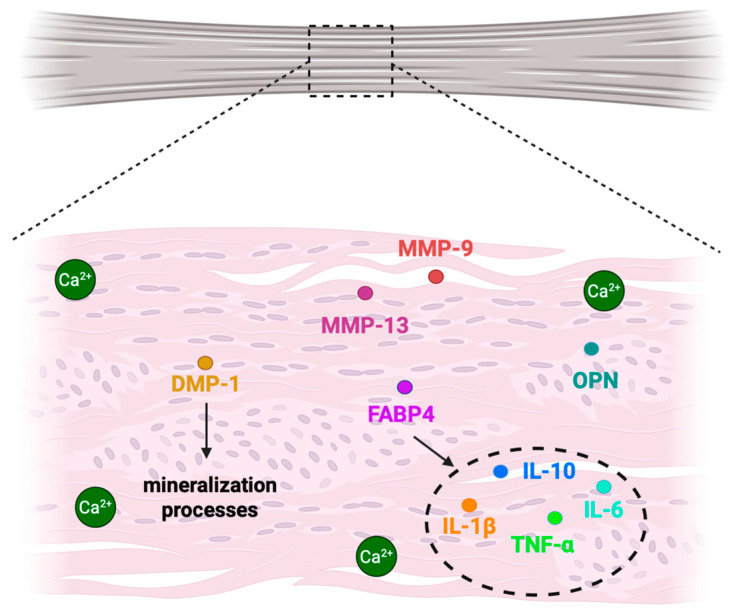
*Several matrix metalloproteinases and inflammatory mediators upregulated in calcified tendon tissue.* Abbreviations: MMP-9 (Metalloproteinase 9), MMP-13 (Metalloproteinase 13), FABP4 (fatty acid binding protein-4), OPN (osteopontin), DMP-1 (dentine matrix protein-1), IL-10 (interleukin 10), IL-1β (interleukin 1 beta), TNF-α (tumor necrosis factor alpha), IL-6 (interleukin 6).

BMP-2 is not the sole BMP implicated in pathological tendon mineralization. Using a collagenase-induced injury model in rat patellar tendons to mimic failed healing, Lui et al. demonstrate ectopic expression of BMP-2, BMP-4, and BMP-7 in chondrocyte-like cells and ossified regions. BMP-2 expression peaked at two weeks post-injury, particularly in fibroblast-like cells within a disorganized tendon matrix and gradually declines between weeks 4 and 16, persisting mainly in ossified regions and associated chondrocyte-like cell populations. In contrast, BMP-4 and BMP-7 exhibit a biphasic expression pattern, with strong expression at two weeks post-injury, a reduction between weeks 4 and 8, and a second increase between weeks 12 and 16 in cartilage- and marrow-like regions, suggesting a more prominent role in regulating the progression and maturation of ectopic ossification [29]. Matrix degradation and inflammation further contribute to disease progression. Several matrix metalloproteinases and inflammatory mediators are markedly upregulated in calcified tendon tissue, including MMP-9 and MMP-13) as well as interleukin-1 receptor antagonist (IL1RN) and osteopontin, with reported increases of up to 25.85-fold and 19.40-fold for MMP-9 and MMP-13, respectively [13]. In parallel, dentine matrix protein-1 (DMP-1), a non-collagenous protein involved in hydroxyapatite nucleation, normally secreted by osteocytes, is detected in calcified regions of tendinopathic tissue, supporting active-matrix mineralization processes [30]. Inflammatory amplification is also driven by fatty acid binding protein-4 (FABP4), a pro-inflammatory adipokine that is upregulated in rotator cuff tendinopathy and promotes cytokine expression in tendon-resident cells through a positive feedback loop involving interleukin-1 beta (IL-1β), interleukin-6 (IL-6), interleukin-10 (IL-10), and tumor necrosis factor alpha (TNF-α) [14] (Figure 2). Additionally, apatite crystals themselves act as inflammatory stimuli by activating macrophages through Nuclear Factor kappa-light-chain-enhancer of activated B cells (NF-kB) signaling and subsequent NOD-, LRR, and Pyrin Domain-Containing Protein 3 (NLRP3) inflammasome activation, leading to IL-1β maturation via caspase-1 [31].

## 3. Discussion

This paper highlights the multifactorial nature of calcific tendinopathy, while also acknowledging significant limitations that future studies must address. Regardless of the contributing factors, the activation of inflammatory mediators appears to be an integral cause of the phenotypic changes observed in tenocytes [27]. Diabetes and thyroid disorders are known to increase the expression of pro-inflammatory cytokines within tendon tissue, and hyperlipidemia is linked to lipid deposition and oxidative stress that further amplify inflammatory signaling. These abnormalities provide different upstream triggers, yet all converge on the same downstream mechanism, namely chronic inflammation. This highlights inflammation as a common pathogenic pathway linking different systemic risk factors to cellular transformation and calcific deposition in tendons [19,21,22]. This concept is further supported by several studies demonstrating that targeted modulation of inflammatory signaling can inhibit calcification and partially restore tendon homeostasis [32,33,34]. The growing understanding of the molecular pathways underlying calcific tendinopathy prompts the development of targeted therapeutic strategies aimed at modulating inflammation, oxidative stress, and aberrant osteogenic differentiation. Reactive oxygen species (ROS) are identified as key drivers of pathological osteogenic differentiation in tendon stem cells. In a murine model, Wang et al. demonstrate that reducing ROS levels inhibit ectopic calcification and promoted organized collagen formation. This is achieved through the localized delivery of a growth hormone-releasing hormone agonist using bovine serum albumin/heparin nanoparticles, enabling sustained release at the inflammatory site and highlighting a promising redox-targeted therapeutic approach [32].

Natural anti-inflammatory compounds also show therapeutic potential. Curcumin, delivered via controlled-release chitosan microspheres, is shown in a rat model to restore collagen fiber organization, reduce inflammatory marker expression, prevent ectopic calcification, and promote tendon regeneration without cytotoxic effects. The sustained local delivery system enhanced curcumin’s bioavailability and therapeutic efficacy, demonstrating benefits both *in vitro* and *in vivo* [33].

Another promising strategy involves targeting Ras-related C3 botulinum toxin substrate 1 (Rac1), a small GTPase implicated in pathological osteogenic differentiation. Pharmacological inhibition of Rac1 using NSC23766 effectively suppresses osteochondral marker expression while restoring tenogenic gene profiles *in vitro*. When locally administered through a chitosan/β-glycerophosphate hydrogel in a rat tendinopathy model, Rac1 inhibition significantly reduces ectopic calcification and enhances tendon regeneration, supporting its potential as a disease-modifying therapy [34].

Although these findings are encouraging, they remain at the preclinical stage, and future research is needed to validate their efficacy and long-term outcomes in humans. Recent studies identify disruption of calcium homeostasis, mediated through the PPP1R3A (Protein Phosphatase 1 Regulatory Subunit 2A)–Piezo1(mechanosensitive ion channel Piezo type 1)–SERCA2 (Sarco/Endoplasmic Reticulum Ca2+-ATPase 2) axis, as an additional central mechanism in the pathogenesis of calcific tendinopathy. In patient tissue and a collagenase-induced rat model, PPP1R3A is downregulated; this alteration is associated with Piezo1 upregulation, SERCA2 reduction, elevated intracellular Ca^2+^, and induction of osteogenic and chondrogenic markers OCN (osteocalcin) and SOX9 (SRY-box transcription factor 9). Tendon-targeted PPP1R3A overexpression reverses these changes and improves matrix organization, and ELAVL1 (embryonic lethal abnormal vision-like protein 1) stabilizes PPP1R3A mRNA, thereby adding a post-transcriptional control point [35]. Despite promising evidence, most of these findings remain limited to animal or *in vitro* models. An additional limitation derives from the narrative nature of this review. Although selected PRISMA items were followed to improve transparency of the search and selection process, a formal systematic review methodology was not fully applicable. In particular, risk-of-bias assessment and quantitative synthesis were not performed because the included studies were highly heterogeneous and largely consisted of preclinical, experimental, or mechanistic investigations. Consequently, conclusions should be interpreted as qualitative synthesis rather than as evidence derived from systematic quantitative aggregation.

Robust human studies are needed not only to validate the role of these molecular pathways and genes, but also to clarify how inflammatory pathways, circadian rhythm disruption, systemic metabolic factors, and genetic variants interact in human tendon biology.

## 4. Materials and Methods

### 4.1. Review Strategy

A narrative literature review was conducted in accordance with selected items of the PRISMA (Preferred Reporting Items for Systematic Reviews and Meta-Analyses) guidelines to ensure methodological rigor and transparency. Because the present work is a narrative review aimed at summarizing mechanistic and translational evidence rather than performing a quantitative synthesis, not all PRISMA items applicable to systematic reviews and meta-analyses were implemented. Specifically, items related to risk-of-bias assessment, quantitative data synthesis, effect-size estimation, and meta-analytic statistical procedures were not applicable, as the included studies were heterogeneous in design, models, and outcomes and no pooled analysis was performed. Therefore, PRISMA guidance was followed only for search transparency, study identification, and selection processes. The main objective was to identify and synthesize evidence regarding the cellular, molecular, and genetic mechanisms contributing to shoulder calcification.

### 4.2. Information Sources and Search Strategy

A comprehensive literature search is performed in the PubMed/MEDLINE, Scopus, and Web of Science databases, including studies published up to 30 September 2025.

No language restrictions are applied, but only studies available in full text are considered.

The research question is structured according to the PICO framework as follows:

- P (Population): adults with forms of shoulder calcification, regardless of specific etiology.
- I (Intervention/Exposure): investigation of cellular, molecular, and genetic factors associated with the calcification process.
- C (Comparison): not applicable.
- O (Outcome): understanding of the cellular, molecular, and genetic mechanisms contributing to shoulder calcification.

The search strategy combines keywords and MeSH terms related to shoulder calcification and the underlying biological mechanisms. The search string, adapts to each database syntax, is as follows:

(“shoulder calcification” OR “calcific tendinitis” OR “calcifying tendinitis” OR “hydroxyapatite deposition” OR “calcific shoulder disease” OR “calcific rotator cuff tendonitis” OR “calcific tendonopathy” OR “shoulder calcium deposits” OR “calcification of the shoulder” OR “calcium deposition in shoulder” OR “shoulder calcific deposits”)
AND
(“cellular mechanism” OR “molecular mechanism” OR “genetic factors” OR “gene expression” OR “inflammatory markers” OR “cytokines” OR “matrix metalloproteinases” OR “MMPs” OR “alkaline phosphatase” OR “tendon cells” OR “fibroblasts” OR “osteogenic differentiation” OR “metabolic disorders” OR “diabetes” OR “thyroid disorders” OR “chondrocytes” OR “osteoblasts”).

### 4.3. Inclusion and Exclusion Criteria

Studies are included if they:

- Involve adult populations affected by shoulder calcification.
- Investigate cellular, molecular, or genetic factors involved in the calcification process.
- Describe pathogenetic mechanisms or biological processes relevant to the disease.

Studies are excluded if they:

- Are not available in full text.
- Are case reports, narrative reviews, or conference abstracts.

### 4.4. Study Selection

Two independent reviewers screen the titles and abstracts of all retrieved records to assess eligibility. Then, potentially relevant articles are reviewed in full text to confirm inclusion.

Discrepancies between reviewers are resolved by discussion or, when necessary, by consulting a third reviewer.

The initial database search identifies 567 records (PubMed = 206, Scopus = 158, Web of Science = 203). After removing 317 duplicates using Rayyan, 250 records remained for screening.

Titles and abstracts are reviewed, and 180 records are excluded for being unrelated to the research question. Seventy articles are retrieved for full-text assessment, of which 45 are excluded for the following reasons: not addressing cellular, molecular, or genetic mechanisms (n = 25), being narrative reviews or case reports (n = 10), or lacking full-text availability (n = 10).

An additional 10 records are identified through citation searching. All are eligible and included in the final synthesis.

**Figure 3:**
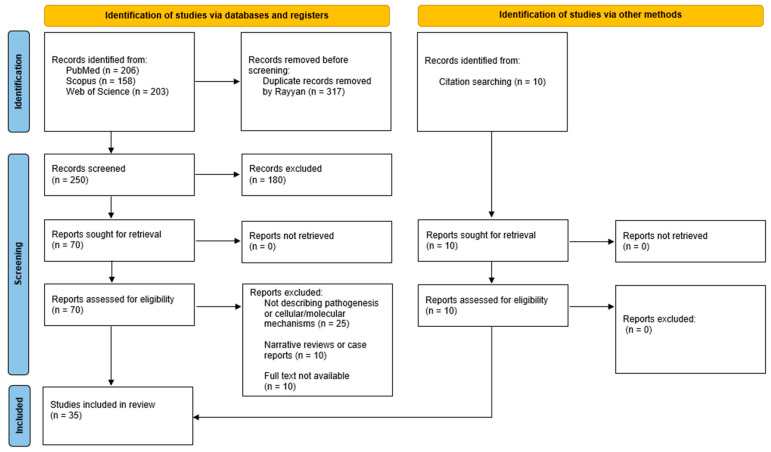
PRISMA 2020 flow diagram for new systematic reviews which included searches of databases and other sources.

In total, 35 studies are included in the narrative review, as illustrated in Figure 3 (PRISMA flow diagram).

## 5. Conclusions

Calcific tendinopathy of the shoulder is a complex and multifactorial disorder shaped by mechanical stress, metabolic and endocrine influences, inflammatory signaling, and genetic regulation. While traditional models, such as Uhthoff’s theory, remain useful for describing disease stages, emerging research highlights a much broader network of molecular and cellular pathways. Experimental interventions targeting inflammation, oxidative stress, and calcium homeostasis show promise in restoring tendon structure in preclinical models, but translation to clinical care remains limited. Some aspects of the calcific tendinopathy of the shoulder remain unclear. It would be interesting to investigate whether there is an interaction between this condition and other shoulder disorders or other anatomical conditions. Unfortunately, the limitation of our work is not having investigated these possible interactions. Current evidence is constrained by small sample sizes, reliance on animal or *in vitro* systems, and the absence of large, well-designed human studies. Future work should aim to identify reliable biomarkers of disease activity and to test targeted therapies in clinical trials and investigate possible interactions between this pathology and other shoulder conditions. Advances in these areas represent the most promising avenue for developing effective and durable treatments for patients suffering from this common and often debilitating condition.

## Figures and Tables

**Table 1:** Main risk factors associated with shoulder calcific tendinopathy.

Risk Factor	Category	Key Findings	Effect Size/Association	Notes	Ref.
Diabetes mellitus	Endocrine/ metabolic	Hyperglycemia-related oxidative stress, AGE accumulation, inflammatory dysregulation, and altered microenvironment promote tendon degeneration and calcification	~27% increased risk over 8 year	Also associated with impaired vascular supply and fibrocartilaginous transformation	[19,20]
Age	Demographic	Risk increases with advancing age	Independent risk factor	Likely reflects cumulative metabolic and degenerative changes	[20,22]
Female sex	Demographic	Higher susceptibility compared with males	Increased risk	Sex-specific biological and hormonal factors implicated	[20,22]
Hyperlipidemia	Metabolic	Markedly increases risk in women	~2-fold increase in women	Suggests sex-dependent interaction	[22]
Hyperthyroiditis	Endocrine	Altered collagen synthesis and extracellular matrix metabolism	Increased prevalence among affected patients	Thyroxine deficiency may impair tendon remodeling	[21]
Occupational repetitive activity	Mechanical/occupational	No significant increase in risk observed	Not significant	Suggests limited role of repetitive movements alone	[23]

**Table 2:** Genetic and molecular conditions associated with shoulder tendon calcification.

Gene	Condition	Pathological Mechanism	Key Features Related to Calcification	Ref.
*ANKH*	Calcium Pyrophosphate Dihydrate Deposition (CPPD)	Increased extracellular PPi transport leading to CPP crystal deposition	Tendon and periarticular calcifications; may mimic or contribute to calcific tendinopathy	[15]
*ANKH*	Chondrocalcinosis 2 (CCAL2)	Upregulation of type X collagen expression; promotion of chondrocyte hypertrophy and cartilage-like differentiation	Early-onset chondrocalcinosis with tendon involvement	[15]
*TNSALP*	Hypophosphatasia	Reduced ALP activity leading to impaired PPi hydrolysis and altered mineralization	Periarticular and ligament calcifications; skeletal abnormalities	[24]
*HGD*	Alkaptonuria	Accumulation of homogentisic acid causing ochronosis and disruption of collagen cross-linking	Degeneration and calcification of tendons and ligaments	[24]
*ABCC6*	Pseudoxanthoma elasticum (PXE)	Reduced extracellular PPi levels and increased tissue-nonspecific ALP activity promoting ectopic mineralization	Rotator cuff calcifications and enthesophytes (up to 78% of patients)	[25]
*RRBFOX2*, *CHD2*, *MBNL1*	Post-transcriptional dysregulation	Cytoplasmic mislocalization of RBFOX2 with loss of nuclear splicing regulation	Altered exon usage in calcified rotator cuff tendons	[26]

## Data Availability

The data that supports the findings of this study are available within the article.
